# Data on mechanical properties of open-cell AlSi10Mg materials and open-cell AlSi10Mg-SiC composites with different pore sizes and strain rates

**DOI:** 10.1016/j.dib.2023.109461

**Published:** 2023-07-29

**Authors:** Mihail Kolev, Ludmil Drenchev, Tatiana Simeonova, Rumen Krastev, Vasil Kavardzhikov

**Affiliations:** a Institute of Metal Science, Equipment and Technologies with Center for Hydro- and Aerodynamics “Acad. A. Balevski” at Bulgarian Academy of Sciences, Bulgaria; b Institute of Mechanics at Bulgarian Academy of Sciences, Bulgaria

**Keywords:** Al-based metal matrix composites, Compression strength characteristics, Stress–strain curve, Energy absorption

## Abstract

This data article describes the stress-strain curves, energy absorption and energy absorption efficiency of open-cell AlSi10Mg materials and open-cell AlSi10Mg-SiC composites with different pore sizes and strain rates. The data were obtained by quasi-static compression loading up to 60% strain at strain rates of 0.01 and 0.001 *s*^−1^ according to ISO 13,314:2011 standard. The data can be used to compare the effects of pore size and strain rate on the compressive properties of the materials. The data are related to the research article entitled “Fabrication, Experimental Investigation and Prediction of Wear Behavior of Open-Cell AlSi10Mg-SiC Composite Materials” (Kolev, M., Drenchev, L., & Petkov, V. (2023). Fabrication, Experimental Investigation and Prediction of Wear Behavior of Open-Cell AlSi10Mg-SiC Composite Materials. Metals, 13(4), 814. MDPI AG. Retrieved from http://dx.doi.org/10.3390/met13040814).

**Specifications Table**

**Table:** 

Subject	Materials Engineering
Specific subject area	Mechanics of composite materials
Type of data	TXT files XLSX files Python programming code (.py) Table Figure
How the data were acquired	The data were acquired by compressing the specimens using a servohydraulic testing machine Zwick-Roell HA-250 equipped with a 250 kN load cell and a piston position sensor. The STM was controlled by computer software that recorded the load-displacement data at a sampling rate of 10 kHz. The specimens were compressed at strain rates of 0.01 and 0.001 *s*^−1^ until reaching 60% strain or failure. The data related to the stress-strain curves, energy absorption and energy absorption efficiency characteristics of the materials were visualized using a Python script.
Data format	Raw Analyzed
Description of data collection	The data were collected by quasi-static compression loading of open-cell AlSi10Mg and AlSi10Mg-SiC materials with different pore sizes and strain rates. This method was chosen to simulate the deformation behavior of the materials under low-speed compression conditions. The specimens were cylindrical with a diameter of 8 mm and a height of 15 mm. They were placed between the lubricated platens of a Zwick-Roell HA250 universal testing machine and were compressed up to 60% strain at strain rates of 0.01 or 0.001 *s*^−1^. The load-displacement data were recorded by the machine's software. The data were normalized by dividing the load by the initial cross-sectional area and the displacement by the initial height of the specimens. The mean normalized load and displacement for each material, pore size, and strain rate were calculated along with their standard deviations and ranges. Experimental data were processed to obtain energy absorption and Energy absorption efficiency both up to a strain of 60%.
Data source location	Institution: Institute of Metal Science, Equipment and Technologies with Center for Hydro- and Aerodynamics “Acad. A. Balevski” at Bulgarian Academy of Sciences. City/Town/Region: Sofia 1574, 67 “Shipchenski prohod” str. Country: Bulgaria. Latitude and longitude: 42.67836627128253, 23.368534487637326
Data accessibility	Repository name: Mendeley Data Data identification number/DOI: 10.17632/9bm255ynwn.1 Direct URL to data: https://data.mendeley.com/datasets/9bm255ynwn Kolev, Mihail; Simeonova, Tatiana; Krastev, Rumen; Kavardzhikov, Vasil (2023), “Mechanical characteristics of open-cell AlSi10Mg materials and open-cell AlSi10Mg-SiC composites with varying pore sizes and strain rates”, Mendeley Data, V1, doi: 10.17632/9bm255ynwn.1
Related research article	Kolev, M., Drenchev, L., & Petkov, V. (2023). Fabrication, Experimental Investigation and Prediction of Wear Behavior of Open-Cell AlSi10Mg-SiC Composite Materials. Metals, 13(4), 814. MDPI AG. Retrieved from http://dx.doi.org/10.3390/met13040814

## Value of the Data

• This data provides comprehensive information on the mechanical behavior of open-cell AlSi10Mg and AlSi10Mg-SiC materials with different pore sizes and strain rates, which are under compression, including numerical data and stress-strain diagrams that are consisted of the elastic region, the plastic plateau and the densification region up to 60% deformation of the tested porous materials.
• The data can be used to compare the effects of pore size and strain rate on the compressive properties of the materials, such as compression strength, plateau stress, plateau end stress, plateau end strain, energy absorption and energy absorption efficiency.
• Researchers and engineers who are interested in designing, optimizing, or evaluating open-cell metal matrix composites for various engineering applications, such as aerospace, automotive, or biomedical devices, can benefit from the data presented here.
• The reported data can be used/reused for further insights and/or development of experiments by performing numerical simulations or analytical models based on the experimental data.
• Advanced data analysis techniques, such as machine learning or artificial intelligence methods, can also be applied to extract more features or patterns from the data.

## 1 Objective

The objective of this dataset is to provide comprehensive information on the compressive properties of open-cell AlSi10Mg material and open-cell AlSi10Mg-SiC composites with different pore sizes and strain rates. These composite materials are novel porous metal composites that have potential applications in high-strength and lightweight structures and that need high friction and wear resistance. The dataset contains the stress-strain curves, energy absorption and energy absorption efficiency characteristics of the materials obtained by quasi-static compression loading. The dataset can be used to compare the effects of pore size and strain rate on the compressive behavior of the materials, as well as to validate numerical models or analytical formulations for predicting their response. The dataset is related to our original research article entitled “Fabrication, Experimental Investigation and Prediction of Wear Behavior of Open-Cell AlSi10Mg-SiC Composite Materials” [1], where we fabricated and characterized the composite material. The dataset adds value to the published article by providing the raw and processed data files of the compressive properties, energy absorption and energy absorption efficiency characteristics, as well as the python script used for data processing and visualization, which can be accessed and reused by other researchers.

## 2 Data Description

The data files contain the average stress-strain curves, energy absorption and energy absorption efficiency characteristics of open-cell AlSi10Mg and AlSi10Mg-SiC materials with different pore sizes and strain rates. The materials were tested under quasi-static compression loading up to 60% strain at strain rates of 0.01 and 0.001 *s*^−1^. The labels of the specimen indicate the following:

• C 0.01 is the average data obtained of the open-cell AlSi10Mg material with pore size of 800 ÷ 1000 µm and compressed with strain rate of 0.01 *s*^−1^;
• C 0.001 is the average data obtained of the open-cell AlSi10Mg material with pore size of 800 ÷ 1000 µm and compressed with strain rate of 0.001 *s*^−1^;
• E 0.01 is the average data obtained of the open-cell AlSi10Mg material with pore size of 1000 ÷ 1200 µm and compressed with strain rate of 0.01 *s*^−1^;
• E 0.001 is the average data obtained of the open-cell AlSi10Mg material with pore size of 1000 ÷ 1200 µm and compressed with strain rate of 0.001 *s*^−1^;
• SC 0.01 is the average data obtained of the open-cell AlSi10Mg-SiC composite with pore size of 800 ÷ 1000 µm and compressed with strain rate of 0.01 *s*^−1^;
• SC 0.001 is the average data obtained of the open-cell AlSi10Mg-SiC composite with pore size of 800 ÷ 1000 µm and compressed with strain rate of 0.001 *s*^−1^;
• SE 0.01 is the average data obtained of the open-cell AlSi10Mg-SiC composite with pore size of 1000 ÷ 1200 µm and compressed with strain rate of 0.01 *s*^−1^;
• SE 0.001 is the average data obtained of the open-cell AlSi10Mg-SiC composite with pore size of 1000 ÷ 1200 µm and compressed with strain rate of 0.01 *s*^−1^.

The raw data files consist of TXT files, which include information for tested specimen and test parameters as: Specimen designation, Pre-load [MPa], Test speed [1/s], Type of compression modulus determination, Material. The numerical data of each tested specimen is presented in 4 columns: Compressive strain [mm], Standard force [N], Time [s] and Work [Nmm]. Those TXT files can be opened by any text editor and are named after tested specimen as follows:

“1C 0,01.TXT”; “2C 0,01.TXT”; “3C 0,01.TXT”: Raw data for C 0.01;
“1C 0,001.TXT”; “2C 0,001.TXT”; “3C 0,001.TXT”: Raw data for C 0.001;
“1E 0,01.TXT; “2E 0,01.TXT”; “3E 0,01.TXT”: Raw data for E 0.01;
“1E 0,001.TXT; 2E 0,001.TXT”; 3E 0,001.TXT”: Raw data for E 0.001;
“1SC 0,01.TXT”; “2SC 0,01.TXT”; “3SC 0,01.TXT”: Raw data for SC 0.01;
“1SC 0,001.TXT”; “2SC 0,001.TXT”; “3SC 0,001.TXT”: Raw data for SC 0.001;
“1SE 0,01.TXT”; “2SE 0,01.TXT”; “3SE 0,01.TXT”: Raw data for SE 0.01;
“1SE 0,001.TXT”; “2SE 0,001.TXT”; “3SE 0,001.TXT”: Raw data for SE 0.001.

The analyzed data files are in XLSX format and can be opened by any spreadsheet software. The first row of each file indicates the material type and its strain rate testing condition. The second row contains the labels of the parameters that are investigated. The third row contains the dimensions of these parameters. The first column contains the strain values (Strain), the second column contains the corresponding stress values, the third contains the corresponding energy absorption values (W), and the fourth contains the corresponding energy absorption efficiency values (We) for each specimen. At the bottom of the file, for each specimen are calculated the plateau stress, plateau end stress and plateau end strain. The data files are named as follows:

“C 0.001 stress-strain curves.xlsx”: Data for C 0.001;
“C 0.01 stress-strain curves.xlsx”: Data for C 0.01;
“E 0.001 stress-strain curves.xlsx”: Data for E 0.001;
“E 0.01 stress-strain curves.xlsx”: Data for E 0.01;
“SC 0.001 stress-strain curves.xlsx”: Data for SC 0.001;
“SC 0.01 stress-strain curves.xlsx”: Data for SC 0.01;
“SE 0.001 stress-strain curves.xlsx”: Data for SE 0.001;
“SE 0.01 stress-strain curves.xlsx”: Data for SE 0.01;
“Average stress-strain curves.xlsx”: Average data for all tested.
“mechanical-properties-all-plots.py”: Python script using the matplotlib library for visualization of all plots from the file “Average stress-strain curves.xlsx”;
“Stress-strain-C-SC.png”: Stored plot file with a PNG extension of the stress-strain of samples C and SC.
“Stress-strain-E-SE.png”: Stored plot file with a PNG extension of the stress-strain of samples E and SE.
“Energy-absorption-C-SC.png”: Stored plot file with a PNG extension of the energy absorption of samples C and SC.
“Energy-absorption-E-SE.png”: Stored plot file with a PNG extension of the energy absorption of samples E and SE.
“Energy-absorption-efficiency-C-SC.png”: Stored plot file with a PNG extension of the energy absorption efficiency of samples C and SC.
“Energy-absorption-efficiency-E-SE.png”: Stored plot file with a PNG extension of the energy absorption efficiency of samples E and SE.

**Fig. 1:**
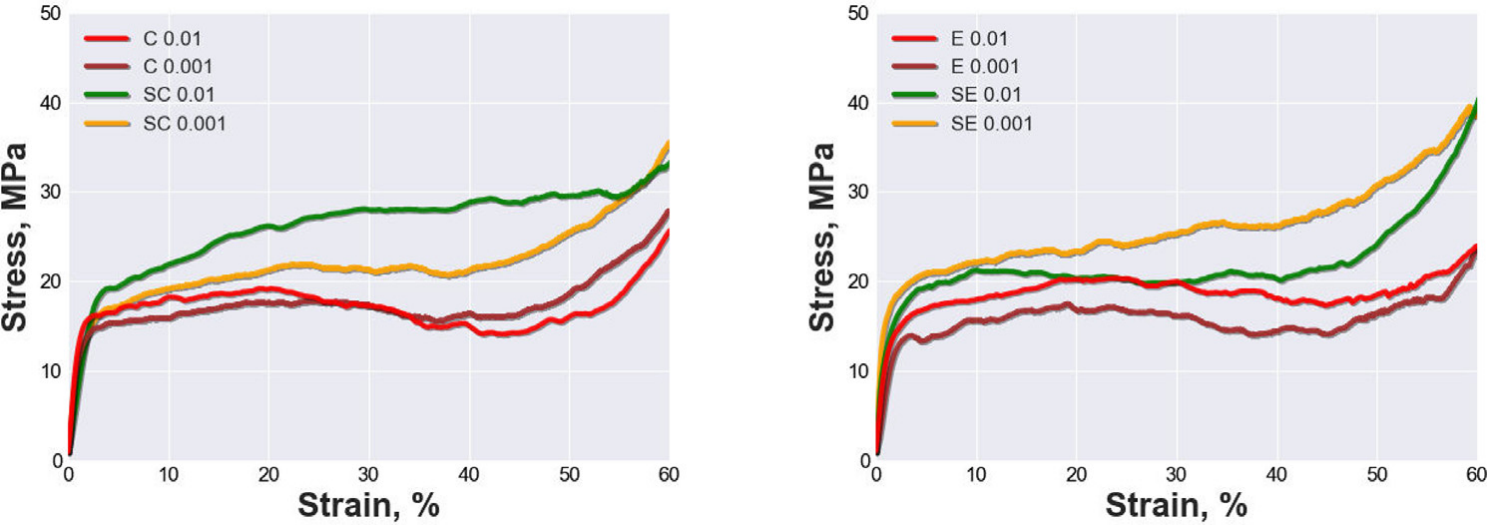
Average stress-strain curves up to 60% strain of all tested materials at strain rates of 0.01 and 0.001 *s*^−1^: (a) AlSi10Mg specimen series C 0.01, C 0.001 and AlSi10Mg-SiC composite specimen series SC 0.01, SC 0.001; (b) AlSi10Mg specimen series E 0.01, E 0.001 and AlSi10Mg-SiC composite specimen series SE 0.01, SE 0.001.

Fig. 1 shows the average stress-strain curves of four types of materials tested at two different strain rates. The curves are based on the average data of three specimens for each material, pore size, and strain rate, which are provided in the data file “Average stress-strain curves.xlsx”. The materials are open-cell AlSi10Mg material and open-cell AlSi10Mg-SiC composite with two different pore size ranges: 800 to 1000 µm and 1000 to 1200 µm. The strain rates are 0.01 and 0.001 *s*^−1^. The curves illustrate the mechanical behavior of the materials under compression, including the elastic region, the plastic plateau and the densification region.

**Fig. 2:**
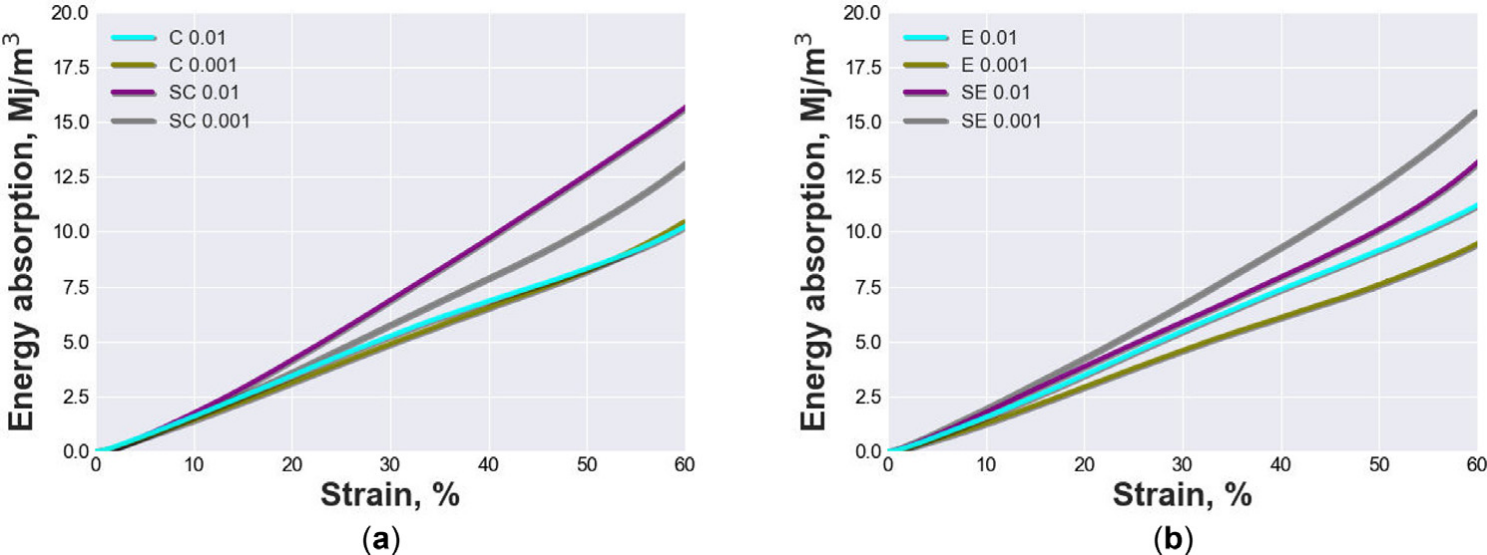
Energy absorption characteristics up to 60% strain of all tested materials at strain rates of 0.01 and 0.001 *s*^−1^: (a) AlSi10Mg specimen series C 0.01, C 0.001 and AlSi10Mg-SiC composite specimen series SC 0.01, SC 0.001; (b) AlSi10Mg specimen series E 0.01, E 0.001 and AlSi10Mg-SiC composite specimen series SE 0.01, SE 0.001.

**Fig. 3:**
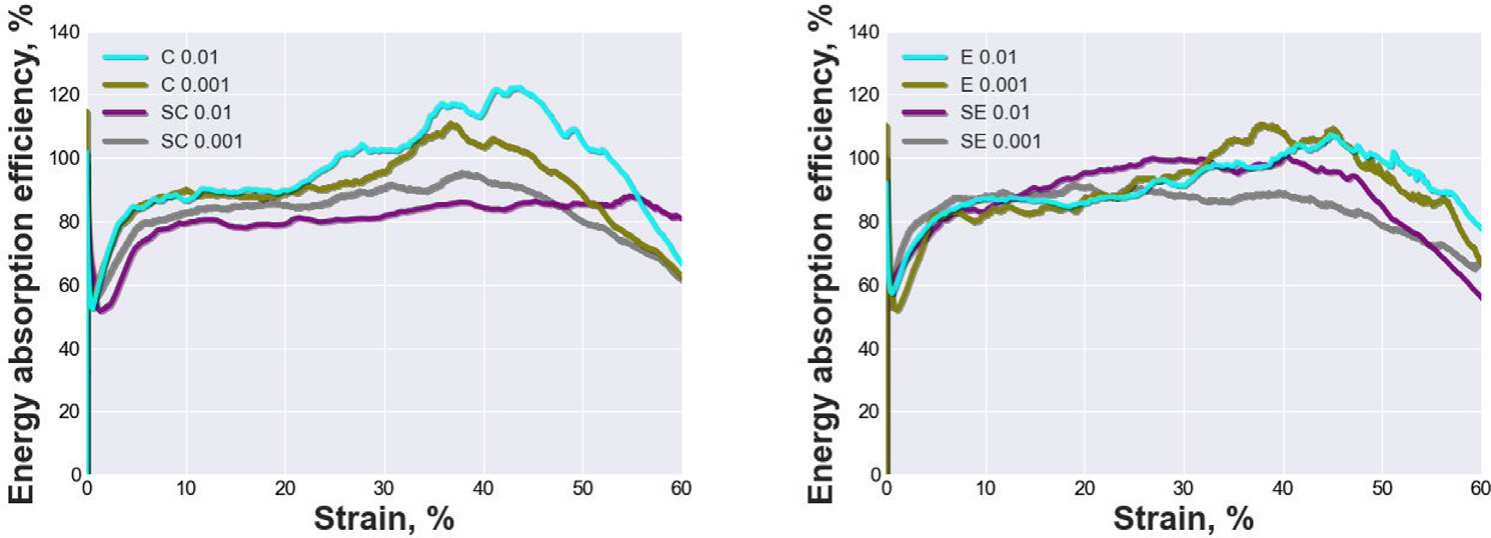
Energy absorption efficiency characteristics up to 60% strain of all tested materials at strain rates of 0.01 and 0.001 *s*^−1^: (a) AlSi10Mg specimen series C 0.01, C 0.001 and AlSi10Mg-SiC composite specimen series SC 0.01, SC 0.001; (b) AlSi10Mg specimen series E 0.01, E 0.001 and AlSi10Mg-SiC composite specimen series SE 0.01, SE 0.001.

Fig. 2 and 3 show the energy absorption and energy absorption efficiency characteristics of open-cell AlSi10Mg and AlSi10Mg-SiC test specimen with different pore sizes and strain rates. The materials were tested under quasi-static compression loading up to 60% strain at strain rates of 0.01 and 0.001 *s*^−1^. Fig. 2(a) compares the energy absorption curves of AlSi10Mg and AlSi10Mg-SiC test specimen with pore sizes ranging from 800 to 1000 µm, while Fig. 2(b) compares the materials with pore sizes ranging from 1000 to 1200 µm. Fig. 3(a) and (b) show the corresponding energy absorption efficiency curves for the same materials and pore sizes. The figures were created by a python script included in the repository “mechanical-properties-all-plots.py”.

The average porosity and relative density of the tested specimens are shown in Table 1.

**Table 1:** Average porosity and relative density of the tested specimens.

Series	Material	Pore size (μm)	Strain rate (s-1)	Average Porosity (%)	Average Relative density
C	AlSi10Mg	800 ÷ 1000	0.01	66	0.34
C	AlSi10Mg	800 ÷ 1000	0.001	66	0.34
SC	AlSi10Mg-SiC	800 ÷ 1000	0.01	64	0.36
SC	AlSi10Mg-SiC	800 ÷ 1000	0.001	64	0.36
E	AlSi10Mg	1000 ÷ 1200	0.01	62	0.38
E	AlSi10Mg	1000 ÷ 1200	0.001	62	0.38
SE	AlSi10Mg-SiC	1000 ÷ 1200	0.01	64	0.36
SE	AlSi10Mg-SiC	1000 ÷ 1200	0.001	63	0.37

## 3 Experimental Design, Materials and Methods

The data presented in this article were collected and analyzed to investigate the compressive properties of open-cell AlSi10Mg and AlSi10Mg-SiC materials with different pore sizes and strain rates. The materials were tested under quasi-static compression loading up to 60% strain at strain rates of 0.01 and 0.001 *s*^−1^. The stress-strain curves, energy absorption and energy absorption efficiency characteristics of the materials were obtained and compared.

The materials used for the experiments were open-cell AlSi10Mg material and open-cell AlSi10Mg-SiC composite with two different pore size ranges: 800 to 1000 µm and 1000 to 1200 µm. The materials were fabricated by liquid-state processing using AlSi10Mg as the base material and SiC particles as the reinforcement. The composite material was fabricated by a liquid-state processing route using the replication method with NaCl as a space holder material mixed with 5 wt.% of SiC particles. The materials and their particle sizes were: NaCl particles with 800 to 1000 µm and 1000 to 1200 µm, and SiC particles with 300 to 400 µm. This method is one of the most commonly employed processes for producing aluminum-based metal matrix composites, according to literature [2,3]. The details of the fabrication process are described in our previous publication [1].

### 3.1 Sample preparation

A lathe was used to shape the specimens from the material into a cylindrical form with a diameter of 8 mm and a height of 15 mm so that sample length to diameter ratio to be between 1 and 2 as it is recommended in ISO 13,314: 2011(E) [4]. The machining process was carefully planned to reduce the possibility of any structural defects that could influence the wear mechanisms. The specimens were machined with a height of 15 mm and a diameter of 8 mm, with aspect ratio 1.875. The machining process was carefully controlled to avoid any structural flaws that could affect the wear mechanisms. The specimens were labeled according to their material type and pore size range as C (800 to 1000 µm), E (1000 to 1200 µm), SC (800 to 1000 µm with SiC) and SE (1000 to 1200 µm with SiC). The average porosity and relative density of tested specimens are shown in Table 1.

### 3.2 Testing equipment

The specimens were tested by a servohydraulic testing machine Zwick-Roell HA-250 equipped with a 250 kN load cell and absolute displacement was measured using the piston position sensor. The equipment was controlled by testXpert III testing software that allows setting the loading conditions, keeping constant strain rate during the entire experiment and recording the load-displacement data.

### 3.3 Testing procedure

The compressive tests were carried out at ambient temperature. Before testing, every specimen is measured at a three measuring points. Test specimens were placed between the two flat platens of the testing equipment, which were lubricated previous to testing and aligned with their axis perpendicular to the loading direction. The specimens were compressed up to 60% deformation at a constant crosshead speed corresponding to the lowest 10^−3^ *s*^−1^ and highest 10^−2^ *s*^−1^ compression strain rates determined in ISO 13,314 standard for compression testing of porous materials [4], in order to determine whether the material is sensitive to strain rate under quasi-static loadings. The load-displacement data were recorded by the testXpert III testing software at an average sampling frequency of minimum 10 Hz. For each tested series of specimens, there were 3 tests.

### 3.4 Data acquisition and data calculation

The load-displacement data of every tested specimen is exported from testXpert III testing software as TXT file. These files contain information about Specimen designation, Pre-load [MPa], Test speed [1/s], Type of compression modulus determination, Material, Compressive strain [mm], Standard force [N], Time [s] and Work [Nmm].

The data is further analyzed by the use of the load and displacement values for each specimen at each time step. In order to calculate the compressive stress, the load values are divided by the specimen cross section area, and the compressive strain in percent is obtained by dividing the displacement values by the specimen height. The plateau stress is determined by calculation of arithmetical mean of all stresses in the interval between 20% and 30% deformation, plateau end stress is calculated by multiplying plateau stress by 1.3 and plateau end strain corresponds to the strain at the plateau end stress.

Experimental data were processed further to obtain energy absorption (W) and Energy absorption efficiency (We) both up to a strain of 60%. The energy absorption (W) and energy absorption efficiency (We) are calculated by following equations:

(1) $$W=\frac{1}{100}\int_{0}^{e_{0}}\sigma \;de,$$

(2) $$W_{e}=\frac{W\left(e\right)}{\sigma_{0}\;\cdot e_{0}}10^{4},$$

where σ_0_ is the compressive stress at the upper limit of the compressive strain (e_0_). The energy absorption integral is solved using trapezoidal approximation. Based on calculated result for every row of raw data the stress-strain curves, energy absorption and energy absorption efficiency graphs are build up.

Data were visualized using a Python script “mechanical-properties-all-plots.py”. The script generated six plots using the matplotlib library to visualize all plots from the file “Average stress-strain curves.xlsx”. The plots were stored as six files with PNG extension. The code files are available as supplementary files or can be accessed from https://doi.org/10.17632/9bm255ynwn.1 (link to repository).

## Ethics Statements

The present work complies with ethical requirements and does not involve human subjects, animal experiments, or any data collected from social media platforms.

## Funding

This research was funded by Bulgarian National Science Fund, Project № КП−06-Н57/20 “Fabrication of new type of self-lubricating antifriction metal matrix composite materials with improved mechanical and tribological properties”.

## CRediT authorship contribution statement

**Mihail Kolev:** Conceptualization, Methodology, Software, Validation, Formal analysis, Investigation, Writing – review & editing, Visualization, Supervision, Project administration, Funding acquisition. **Ludmil Drenchev:** Conceptualization, Methodology. **Tatiana Simeonova:** Conceptualization, Methodology, Software, Validation, Formal analysis, Investigation, Visualization, Project administration. **Rumen Krastev:** Methodology, Software, Validation, Formal analysis, Investigation. **Vasil Kavardzhikov:** Methodology, Formal analysis, Investigation.

## Data Availability

Mechanical characteristics of open-cell AlSi10Mg materials and open-cell AlSi10Mg-SiC composites with varying pore sizes and strain rates (Original data) (Mendeley Data).
